# To Correspondents

**Published:** 1867-10

**Authors:** 


***To Correspondents.***

A large number of original communications are on file, for which thanks are hereby tendered. The concluding pages of the treatise on the *Endoscope* will be given in the next (November) number, after which we shall have more room for favors. It is impossible for the Editor to reply personally to the large number of letters addressed to him—if the task was undertaken all other business would have to be neglected.

Professional opinions, in cases of disease, are part of the

Editor's stock in trade, as one of the fraternity, and he cannot retail them without the *quid pro quo.* This latter hint may, perhaps, save some manuscript. One word further—articles which have been published in other journals or books have survived the period of their interest for this journal. Be it remembered that the case is rare and exceptional where an article is *reproduced* or regenerated in these pages.

